# Current Progress on MicroRNA-Based Gene Delivery in the Treatment of Osteoporosis and Osteoporotic Fracture

**DOI:** 10.1155/2019/6782653

**Published:** 2019-03-06

**Authors:** Xi Sun, Qi Guo, Wenhua Wei, Stephen Robertson, Ying Yuan, Xianghang Luo

**Affiliations:** ^1^Department of Endocrinology, The Third Xiangya Hospital of Central South University, 138# Tongzipo Road, Changsha, Hunan 410007, China; ^2^Department of Endocrinology, Endocrinology Research Center, Xiangya Hospital of Central South University, 87# Xiangya Road, Changsha, Hunan 410008, China; ^3^Department of Women's and Children's Health, Dunedin School of Medicine, University of Otago, Dunedin 9016, New Zealand

## Abstract

Emerging evidence demonstrates that microRNAs, as important endogenous posttranscriptional regulators, are essential for bone remodeling and regeneration. Undoubtedly, microRNA-based gene therapies show great potential to become novel approaches against bone-related diseases, including osteoporosis and associated fractures. The major obstacles for continued advancement of microRNA-based therapies in clinical application include their poor *in vivo* stability, nonspecific biodistribution, and unwanted side effects. Appropriate chemical modifications and delivery vectors, which improve the biological performance and potency of microRNA-based drugs, hold the key to translating miRNA technologies into clinical practice. Thus, this review summarizes the current attempts and existing deficiencies of chemical modifications and delivery systems applied in microRNA-based therapies for osteoporosis and osteoporotic fractures to inform further explorations.

## 1. Introduction

Bone, as the only support organ of an organism, is constantly modeled and remodeled to better fulfill its function throughout life. Through precisely mediating the balance of different bone-related cells, bone has a conspicuous capacity to spontaneously regenerate, and it can repair minor defects by itself [1]. However, in regard to a critically sized osseous deficiency, the repair is insufficient and sluggish [2], especially for osteoporosis patients who have disordered bone metabolism [3]. Osteoporosis is an age-related bone disease characterized by the loss of bone mass, impairment of bone microarchitecture, decrease in bone strength, and thus increased risk of fracture [4]. Bone fractures in elderly patients with osteoporosis are difficult to heal completely and easy to form nonunion or delayed union even with excellent clinical interventions [3, 5]. In fact, osteoporotic fractures have become one of the major factors causing disability and mortality in elderly people; for example, of the patients suffering from osteoporotic hip fracture, 20% die within one year and additional ~50% become physically disabled with greatly reduced quality of life [6, 7].

Antiosteoporotic therapies are classified into two categories: antiresorptive drugs which inhibit bone resorption by disturbing the biological behavior of osteoclasts and anabolic treatments which promote bone formation through increasing the bone remodeling rate [8]. Despite the outstanding effect against osteoporosis, the side effects such as gastrointestinal intolerability [9], osteonecrosis [10], oversuppression of bone turnover [11], thromboembolic disease [12], and increased risk of osteosarcoma [13] and ovarian/endometrial/breast cancers [14] limit the long-term use of these antiosteoporotic drugs. Overall, there is still great demand for the development of novel safe and more efficacious antiosteoporotic drugs characterized by a larger therapeutic window with reduced side effects.

Bone regeneration medicines hold promise in treating complicated bone fractures via restoring normal functions of damaged cells or tissues. Cytokines and growth factors, such as bone morphogenetic proteins (BMPs), are widely used to augment the osteoinduction of regeneration materials [15]. However, the use of recombinant osteogenic proteins is constrained in clinical settings due to their poor stability, high cost, and short half-life. Moreover, compared with the normal concentration in bone, the doses of recombinant human BMP-2 needed for bone regeneration are much higher, which may bring about osteolysis or ectopic bone formation at the site of implantation [16]. Thus, more proper alternatives are needed to ameliorate these bone regeneration materials.

MicroRNAs (miRNAs) are a class of single-stranded noncoding RNAs, ~22 nucleotides in length, which are widely expressed among eukaryotes [17, 18]. During the past two decades, miRNA has demonstrated unprecedented therapeutic potential for osteoporosis and refractory osteoporotic bone defects due to its important role in bone metabolism through regulating the proliferation, differentiation, and function of bone cells. Unfortunately, there are two major barriers to translating miRNA-based therapeutics into clinical settings, the limited half-life of naked synthetic oligonucleotides due to degradation by abundant nucleases in the blood stream or inside cells and the poor capacity to penetrate the host cell membranes and selectively distribute the desired tissues or cells [19]. To overcome the innate deficiency of therapeutic miRNA molecules, two different approaches have been recommended: introducing modifications that optimize oligonucleotide chemistry and using delivery systems that protect RNAs from nucleases and allow endosomal escape. Small interfering RNA (siRNA) is another species of noncoding RNAs. miRNAs and siRNAs belong to the RNA interference (RNAi) effectors and have similar structures and functions. Recently, patisiran, a double-stranded siRNA, has been approved in the USA and EU for treating the polyneuropathy of hereditary transthyretin-mediated amyloidosis (hATTR) in adults [20]. Antisense oligonucleotides are designed to modulate RNA function, including blocking miRNA function, in mammalian cells. Several modified antisense oligonucleotides, such as nusinersen [21], defibrotide [22], and eteplirsen [23], have also been used in clinical practice. Hence, miRNA-based therapeutics will be approved for use in the clinic after the deep research and rational design not long in the future. This review will concentrate on the state-of-the-art of miRNA chemical modifications and miRNA delivery systems and highlight their prospects for the treatment of osteoporosis and osteoporotic fractures.

## 2. Biology of Bone Remodeling and Bone Regeneration

**Figure 1:**
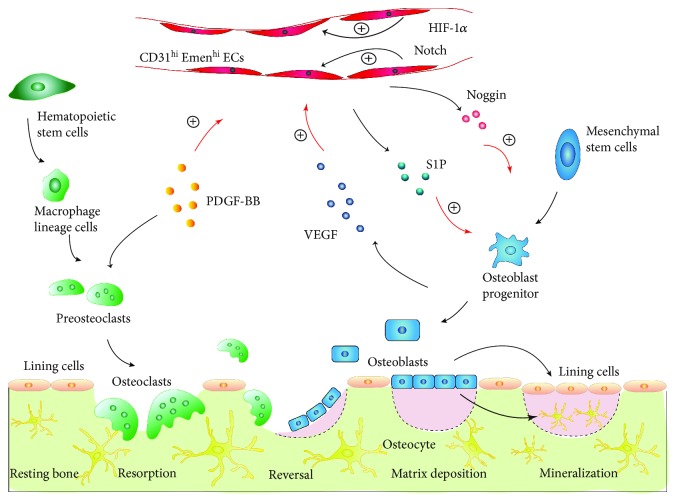
Mechanism of bone remodeling. Under basal conditions, the bone surface is covered by bone lining cells. Local microdamage will recruit hematopoietic stem cell-derived preosteoclasts to differentiate into mature osteoclasts. Mature osteoclasts absorb bone mineral and matrix. After the resorption phase, MSCs are recruited to differentiate into mature osteoblasts, which secrete and mineralize new bone matrix. Once the microdamage is restored, mature osteoblasts will terminally differentiate into either bone lining cells or osteocytes. Type H vessels also participate in the bone remodeling process, which help form a regulation loop comprising preosteoclasts, osteoblasts, and CD31^hi^Emcn^hi^ ECs. PDGF-BB secreted by preosteoclasts, as well as activated endothelial Notch and HIF-1*α* signals, induces CD31^hi^Emcn^hi^ EC proliferation and vessel growth in bone. Notch activity stimulates the expression of endothelial Noggin, whereas PDGF-BB induces CD31^hi^Emcn^hi^ ECs to secrete S1P. Then, increased Noggin and S1P promote osteoblast differentiation and thereby osteogenesis. Meanwhile, the augmented osteoblast numbers secrete vascular endothelial growth factor (VEGF) and positively regulate type H vessel proliferation.

Bone is a dynamic tissue that is continuously turned over throughout life. Normal bone homeostasis depends on a self-renewal mode named bone remodeling which is mainly maintained by the balance between osteoclastic bone resorption and osteoblastic bone formation [24]. In general, the process of bone remodeling can be divided into three stages. Osteoclasts are multinucleated cells originating from hematopoietic stem cells. Cytokines released at the bone remodeling site in stage one recruit osteoclasts to the bone surface which induces osteoclastic bone resorption. The dissolved bone extracellular matrix (ECM) then delivers regulators to recruit bone marrow mesenchymal stem cells (BMSCs) to the bone remodeling site, inducing the osteoblast differentiation of BMSCs, and thus the bone resorption turns into bone formation. Finally, with maturation and mineralization of bone ECM, the osteoblasts either undergo apoptosis or differentiate into bone surface lining cells or osteocytes which are embedded in mature ECM [25, 26]. Despite the potential to differentiate into osteoblasts, BMSCs can also differentiate into adipocytes [27]. Bone is a highly vascularized tissue. In addition to its canonical roles in transporting nutrients oxygen and biowaste production, the bone vasculature can function as a communication network between osteoblasts and osteoclasts, giving a complementary concept of bone remodeling (Figure 1). In 2014, Kusumbe and his colleges [28] identified a specific bone vessel subtype (type H vessel) in mice which was strongly positive for CD31 and endomucin (CD31^hi^Emcn^hi^) and was specifically located in the endosteum and metaphysis of mice. CD31^hi^Emcn^hi^ EC proliferation and endothelial Noggin expression in bone promoted by activating Notch and hypoxia-inducible factor 1 alpha (HIF-1a) signals could induce proliferation and differentiation of perivascular osteoprogenitors and then promote osteogenesis [29]. The platelet-derived growth factor-BB (PDGF-BB) secreted by preosteoclasts increased CD31^hi^Emcn^hi^ vessel number and stimulated bone formation during bone modeling and remodeling [30]. In some conditions, such as aging, menopause, and the use of chronic glucocorticoid (GC) therapy, osteoclasts will be excessively activated, osteoblasts will be functionally suppressed, BMSCs will switch from osteogenesis towards adipogenesis, or the formation and function of bone vasculatures will be impaired, ultimately leading to osteoporosis.

Intramembranous ossification and endochondral ossification are the two forms of bone fracture healing, which involve many well-orchestrated events. At the early phase, MSCs are recruited to the healing site and differentiated into fibrocytes, chondrocytes, or osteoblasts; at the mid-phase, a hard callus and ECM are formed, along with angiogenesis and revascularization; and at the late phase, the callus should be continuously remodeled to meet the biomechanical and biological demand of new bone [31]. The repair of osteoporotic bone fractures is harder to be implemented than that of normal bone fractures because a number of determining factors are disordered, including decreased levels of MSCs and angiogenesis, impaired osteoblast differentiation, and delayed formation and revascularization of the callus.

The disturbed factors during the occurrence of osteoporosis and the impeded healing of osteoporotic fractures are high-potential therapeutic targets. miRNAs have been proven to participate in modulations of almost all of the procedures described above. Thus, interventions for changing miRNA expression show great application and research values for the treatment of osteoporosis and enhancement of bone healing for osteoporotic fractures.

## 3. miRNA-Based Therapeutic Approaches

Depending on the expression modes of the target miRNA, miRNA-based therapeutics can be divided into two categories: gain-of-function and loss-of-function [32]. Gain-of-function means restoring the expression of miRNA suppressed in diseases, while loss-of-function implies blocking the activity of overexpressed miRNA during the pathological process.

Gain-of-function strategies mainly depend on either miRNA mimics or on viral vectors to overexpress miRNAs [33]. miRNA mimics are always synthetic nonnatural double-stranded small RNA molecules functioning similarly to natural miRNAs [34]. This double-stranded RNA molecule mimicking an endogenous miRNA has its “seed region” at the 5′-end which preferentially pairs with selected sites on the 3′ untranslated regions (3′UTR) of the target mRNA and produces posttranscriptional repression of the gene once introduced into cells. miRNA mimics can be designed either to target a single mRNA through binding to the unique sequence in 3′UTR of the target gene distinct from other genes in order to avoid unnecessary downregulation of nonspecific genes [35] or to group multiple miRNA units for different mRNAs in order to achieve linked multigene repression and augment the modulation capacity of a specific physiological process [36]. Viral vector-mediated miRNA overexpression is sustained and stable. This viral vector always comprises a DNA plasmid that contains a miRNA precursor or mature miRNA region, a promoter region, and an antibiotic resistance region [33, 34, 37].

The regulating effects of loss-of-function can be achieved by three patterns: miRNA sponges, miRNA masks, and anti-miRNA oligonucleotides (anti-miRs).

miRNA sponges are transcripts with multiple binding sites, and they function as a decoy to sequester miRNAs from their endogenous targets. They can be designed to target a single specific miRNA or a whole family of related miRNAs sharing a common seed region [38–40]. A strong available tissue-specific promoter is always used to maximize sponge expression in certain cell types of interest and to reduce the influence on unintended cells [41]. Although sponge technology has many advantages for exploring miRNA function, its clinical application for bone deceases is facing tremendous challenges, i.e., cell cytotoxicity, off-target effects, and genomic integration of foreign genes.

Compared with miRNA sponges and other miRNA antagonists, the inhibitory action of miRNA masks is more selective and gene-specific [42]. miRNA masks are single-stranded modified oligonucleotides which are completely base-pairing with the proleptic miRNA-binding site in the 3′UTR of the target mRNA [43]. Despite the fact that each single miRNA may regulate many protein-coding genes, the miRNA mask suppresses the only one gene through selectively blocking the interaction between its target miRNA and the unique mRNA of interest [35].

Anti-miRs are single-stranded synthetic oligonucleotides designed to bind directly to the endogenous miRNAs of interest and block the miRNA-induced repression of mRNA translation through disruption of the miRISC complex [34]. Anti-miRs are the most commonly used interference reagents of miRNA, and they have tremendous potential for clinical translation [33]. However, the use of unmodified RNAs *in vivo* faces many challenges such as the limited half-life, frail filtration by the renal route, removal by phagocytic immune cells, nonspecific biodistribution, and inefficient endocytosis by target cells [44]. Different chemical modifications have been developed in anti-miRs to optimize their biological properties and make them more suitable for clinical application.

## 4. Chemical Modifications for Anti-miRs

The first antisense oligonucleotides used for miRNA silencing were unmodified DNA sequences that were rapidly degraded by endogenous nucleases in biological environments and failed to act on target miRNA efficiently *in vivo* [45]. Chemical modifications introduced in the sugar ring and/or the backbone significantly enhance the performance of anti-miRs through augmenting nuclease resistance, enhancing binding affinity, and improving cellular uptake [46].

Phosphorothioate (PS) linkage is the first reported and most commonly used internucleotide modification on anti-miRs with a nonbridging oxygen of the phosphodiester (PO) bond substituted by a sulfur [46, 47]. PS linkages delay plasma clearance and enhance cellular uptake due to their nonspecific binding to serum albumin and membrane proteins, making PS-modified anti-miRs suitable for *in vivo* delivery [48–50]. However, the overextended PS modification in an anti-miR displays increased tendency to bind to off-target proteins, causing toxicity to normal cells. Moreover, despite the excellent nuclease resistance, PS oligonucleotides present decreased binding affinity to target RNA [51]. Thus, more rational designs, like combining other substitutions, must be taken into account to further enhance the potency of PS-modified anti-miRs.

AntagomiRs (anti-miRs with cholesterol-conjugation, 2′-O-methyl-modification (2′-OMe) and a terminal PS linkage) are the first miRNA inhibitors demonstrated to work in mammals [52]. They have enhanced transmembrane capability, and they have been used *in vivo* to target bone-specific miRNAs and alter bone metabolism. Tail vein injection of antagomiR-148a significantly inhibited miR-148a expression in bone, and the suppressive effect lasted for 3 weeks [53]. In addition, the blocking of miR-148a in bone induced by antagomiR-148a suppressed bone resorption and increased bone mass in OVX mice. AntagomiR-31a-5p was used to silence miR-31a-5p in BMSCs and osteoclasts through periosteal injection into the femoral bone marrow cavity, stimulating bone formation and reducing osteoclastogenesis in aged rats [54]. miR-103 was the first reported mechanosensitive miRNA to inhibit bone formation through targeting Runx2, and pretreatment with antagomiR-103a partly counteracted the bone loss in hindlimb-unloaded (HU) mice [55]. On the whole, antagomiRs are potentially promising therapeutic agents for postmenopausal, age-related, and secondary osteoporosis through inhibiting the pathologically overexpressed miRNAs.

Peptide nucleic acids (PNAs) are synthetic uncharged oligonucleotide analogues with the N-(2-aminoethyl)-glycine polyamide structure replacing the entire sugar-phosphate backbone [56]. When used as anti-miRs, PNAs hybridize to complement nucleic acid sequences of target miRNAs with extraordinarily high affinity and sequence specificity [57]. This kind of hybridization obeys the natural Watson–Crick hydrogen-bonding rules [58]. In addition, PNAs combined with cell-penetrating peptides (CPPs) have strengthened cell penetration ability and enhanced target tissue specificity [46].

Locked nucleic acids (LNAs), along with 2′-OMe, 2′-O-methoxyethyl (2′-MOE), and 2′-fluoro (2′-F), are commonly investigated sugar modifications. LNAs lock the structure into a 3′-endo sugar conformation effectively by tethering the 2′-O to the 4′-C via a methylene bridge [59]. Anti-miRs modified by LNAs have a very strong binding affinity, a high melting temperature, and strong nuclease resistance [60]. A phase II clinical trial showed that five weekly injections of miravirsen, an LNA-modified phosphorothioate oligonucleotide specially suppressing miR-122, led to a prolonged and dose-dependent decrease in serum hepatitis C virus RNA, cholesterol, and alanine aminotransferase levels in chronic hepatitis C patients [61]. No long-term safety problems, even up to 35 months following therapy, were observed among the chronic hepatitis C patients [62]. These discoveries are excellent evidence demonstrating that artificial synthetic anti-miRs designed through concern and intelligent rules have extraordinary potential for clinical use to treat diseases, including osteoporosis and osteoporotic fractures.

The examples described above demonstrate the tremendous efforts to discover chemical modifications that promote the performance of anti-miRs *in vivo* and *in vitro* through enhancing nuclease resistance, binding affinity, and cellular uptake. LNA-anti-miRs combined with other modifications even show great potential for clinical therapeutic application.

## 5. miRNA Delivery Systems for Osteoporosis and Osteoporosis Fractures

Although considerable progress of chemical modifications has been made to promote bioactivity while decreasing the side effects of therapeutic miRNA molecules, many obstacles still need to be solved to make them more powerful and suitable for clinical translation. First, the ability of recognition and loading into Argonaute proteins (AGOs) may be suppressed when excessive stabilizing modifications are introduced in miRNA mimics. Second, the high dose (i.e., ~80 mg/kg [55, 63, 64] for antagomiRs) required for effective inhibition of the majority of therapeutic miRNA molecules *in vivo* increases the risk of severe toxic side effects. The last and most critical obstacle is the limited tissue-specific distribution and poor cellular uptake of chemically modified oligonucleotides when administered *in vivo* without a carrier. Thus, the effective and safe delivery vectors for therapeutic miRNA modulators hold the key to translating miRNA technologies to the treatment of bone diseases.

miRNA delivery systems can be divided into systemic or scaffold-mediated delivery. Systemic delivery systems mainly include viral and nonviral vectors which deliver miRNA modulators *in vivo* directly with high transfection efficiency and good biocompatibility [34]. These vectors are suitable for miRNA replacement/inhibition therapies of osteoporosis, which is a chronic disease and always treated by systematic administrations. However, other than systematic administrations, the treatment of osteoporotic fractures with critical-size bone defects requires a high local and sustained concentration of these synthesized oligonucleotides for bone regeneration. Scaffold-mediated delivery meets this requirement by loading or immobilizing artificial miRNA modulators in or onto the biomaterials for bone tissue engineering.

### 5.1. Viral Vector-Based Systemic miRNA Delivery Systems

Viral vector-based gene delivery is one of the most common gene transfer techniques. Frequently used viral vectors include retrovirus, lentivirus, adeno-associated virus (AAV), baculovirus, and others. Viral vectors that encode RNA molecules can transfect the majority of cell types with high efficiency, avoid the decrease in the concentration of exogenous miRNA modulators with cell division, and thereby prolong the effect of miRNA replacement or inhibition *in vivo* [65, 66].

Retroviruses are a class of lipid-enveloped viruses containing two copies of linear, nonsegmented, single-stranded RNA molecules. In the cytoplasm, the viral RNA is reverse-transcribed into double-stranded DNA which is subsequently transported into the nucleus and integrated into one of the host chromosomes [67–69]. The inhibition of endogenous miR-204 and its homolog miR-211 elicited by retroviral “sponge” vectors containing two copies of miR-204 complementary oligos improved osteoblast differentiation and impaired adipocyte formation of mesenchymal progenitor cell lines and BMSCs [70]. Generally, retroviral vectors (RVs) are reformed to become replication-defective by removing the trans-acting viral genes that are necessary for viral gene expression and replication. It is very important for the clinical use of RVs, because their replicators can infect other cells and bring pathogenic effects [66]. Despite the fact that RVs have been widely utilized in life sciences research over several decades, there are few studies about *in vivo* microRNA delivery for treating osteoporosis or osteoporotic fracture by RVs. The inability of transducing nondividing cells and the dysfunction of host cells caused by genome integration at an undesired location limit the development of RVs at least to some extent.

Lentivirus belongs to the retrovirus family. Compared with RVs, lentivirus vectors (LVs) are more promising tools for gene therapy platforms that target quiescent cell types because they can translocate an intact nuclear membrane across the nuclear pore [71]. Moreover, the characteristic that LVs have additional selection criteria for integration, preferring integrating within introns of active transcriptional units, reduces the likelihood of insertional oncogenesis [72, 73]. Thus, LVs are more frequently used as *in vivo* miRNA delivery vectors to improve bone metabolism and promote bone regeneration. Wang et al. [74] constructed lentivirus-mediated miR-29a precursor expression vectors which attenuated the adverse effects of GC on bone microstructure and bone biomechanical properties via tail vein injection [74]. Overexpression of miR-429 [75] through directly injecting lentivirus into the subcutaneous region of a local fracture accelerated bone formation and remodeling and promoted fracture healing compared with the control group.

Baculoviruses are a group of enveloped, insect-pathogenic viruses that contain a double-stranded circular DNA genome [76]. Baculovirus vectors possess many advantages for *in vivo* gene delivery. They can transfer genes into many mammalian cell types with high efficiency [77]. They lack the capabilities of replication and gene integration in mammalian cells and thereby have an inherently low-risk biosafety profile [78]. Furthermore, baculoviruses are not associated with human diseases, and baculoviral DNAs degrade in mammalian cells over time. Recent studies reported that baculoviruses can encode miRNAs to manipulate the expression regulation of host genes [79]. In addition to these properties, the high transduction efficiencies (>95%) for BMSCs [80] and ASCs [81, 82] make baculovirus vectors a promising delivery tool for miRNA-based gene therapy of osteoporosis and osteoporotic fractures. In an osteoporotic rat model with a critical-size bone defect at the femoral metaphysis, allotransplantation of the baculovirus-engineered OVX-BMSCs (BMSCs isolated from OVX rats) that express miR-214 sponges, with or without BMP2 expression, healed the defect and improved the bone quality, whereas implanting the OVX-BMSCs ectopically expressing BMP2 failed to do that [83]. Another baculovirus-based delivery system coexpressing miR-148b/BMP2 was applied for genetic modification of human adipocyte-derived stem cells (hASCs) [84]. Implantation of these modified hASCs significantly accelerated the bone remodeling and healing of the critical-size calvarial defects in nude mice, filling ≈94% of the defect area and ≈89% of the defect volume with native calvaria-like flat bone in 12 weeks.

AAV, one of the smallest viruses, contains an ~4.7 kb linear single-stranded DNA genome [85]. AAV can infect dividing and nondividing cells with coinfection of a helper virus (such as adenoviruses and herpesviruses) and achieve efficient and sustained gene transfer [86]. AAV vectors have been used in various preclinical animal studies based on a number of advantages, such as nonpathogenicity in humans, wide and promiscuous tropism, and relatively low immunogenicity [87]. Because of the small size, AAV vectors are particularly suitable for miRNA-based gene delivery *in vivo*. Currently, AAV vectors have emerged as the most promising tool for clinical applications. In 2012, Glybera®, an AAV vector designed to treat lipoprotein lipase deficiency through expressing lipoprotein lipase in muscle, was approved as the first gene therapy product in the European Union [88].

Other virus-based vectors can also be used for miRNA delivery in bone-related cells. Yao et al. [89] developed a miRNA delivery system based on bacteriophage MS2 virus-like particles (MS2 VLPs). This delivery system has been proven to be effective in infecting human peripheral blood mononuclear cells (PBMCs), inducing overexpression of miR-146a, and thereby suppressing the differentiation and function of osteoclasts.

The flourishing studies have shown that engineered viruses are excellent materials for miRNA delivery into bone tissues. Despite the promising results of the preclinical trials on viral vectors, their terrible and possibly lethal side effects including insertional mutagenesis that may cause cancer and immune responses in the host have limited their clinical application [90]. These defects pressure scientists to find less pathogenic and immunogenic options.

### 5.2. Nonviral Vector-Based Systemic miRNA Delivery Systems

Although viral vectors have higher transfection efficiency in most mammalian cells (especially in primary cells), nonviral vectors have attracted increasing attention, and they show great potential for clinical translation. Nonviral vectors mainly include lipid-based vectors and polymer-based vectors. They are inexpensive to manufacture at Good Manufacturing Practice and easy to modify, have lower immunogenicity and unrestricted gene material size, and impose a lower degree of genetic perturbation [91]. Due to these features, nonviral vectors have been widely applied for miRNA and siRNA delivery *in vitro* and *in vivo*. siRNAs are structurally and functionally similar to miRNAs. Thus, investigations of synthetic nonviral vectors for siRNA provide inspiration for scientists to design suitable nonviral vectors for miRNA therapeutic delivery [92].

#### 5.2.1. Lipid-Based Nonviral Vectors

The most commonly used nonviral delivery systems are lipid-based nanocarriers, with modifications such as PEGylation or ionization to reduce cytotoxicity and nonspecific uptake [19, 91]. Many commercially available cationic lipid-based products can be selected, such as Lipofectamine® [93], Invivofectamine® [94], Oligofectamine™ [95], and siPORT™ NeoFX™ [96]. BMSC sheets have been reported as a promising regenerative material for fast and high-quality bone repair [97, 98]. By using a properly adapted and optimized Lipofectamine 2000-based formulation, Yan et al. successfully delivered anti-miR-138 into the BMSC sheets with high efficiency [93]. After 8 weeks of *in vivo* implantation, remarkable bone formation ability of the anti-miR-138-modified BMSC sheets was shown by massive regenerated bone with good vascularization, indicating great clinical significance for bone defect repair and regeneration. Tail vein injection of Invivofectamine 3.0 encapsulating a miR-451a mimic significantly upregulated the expression of miR-451a in femur bone extracts, improved osteoblastogenesis mineralization, increased bone strength, and enhanced bone mass of OVX mice [94]. In general, lipid-based particles tend to be distributed in the liver, lungs, and spleen [19]. To overcome the passive accumulation, the nanocarriers are designed to attach to tissue target agents, which significantly increase the specific biodistribution and reduce the unwanted effects on nontarget organs.

Six repetitive sequences of aspartate, serine, and serine ((AspSerSer)6) display a clear tendency to bind to osteoblast-mediated mineralizing nodules and amorphous calcium phosphate *in vitro*. Based on this fact, Zhang et al. [99] developed a delivery system selectively approaching bone-formation surfaces comprising 1,2-dioleoyl-3-trimethylammonium-propane- (DOTAP-) based liposome (approved by the U.S. Food and Drug Administration for clinical trials, NCT00059605) linked with (AspSerSer)6 in 2012. Through intraperitoneal injection of this (AspSerSer)6-liposome-containing casein kinase-2-interacting protein-1 (Plekho1) siRNA, both healthy and osteoporotic rats showed significantly improved bone formation, promoted bone microarchitecture, and increased bone mass [99]. Afterwards, studies successfully delivered antagomiR-214 [100] and agomiR-33-5p [101] into osteoblasts by the (AspSerSer)6-liposome delivery system *in vivo*. Osteoblast-specific downregulation of miR-214 levels counteracted the decrease in bone formation in *Bglap2-*miR-214 transgenic, OVX, and HU mice [100], whereas supplementing miR-33-5p in osteoblasts attenuated osteopenia development induced by mechanical unloading in mice [101].

In contrast, eight repeating sequences of aspartate (D-Asp8) prefer to bind to highly crystallized hydroxyapatite (HA), which is the physicochemistry characteristic of bone-resorption surfaces occupied by osteoclasts and osteoclast precursors [102]. Through linking a modified D-Asp8 peptide with a DOTAP-based liposome, Liu et al. [103] produced a delivery system specifically targeting bone resorption surfaces. Moreover, D-Asp8-liposome-antagomiR-148a demonstrated the ability to concentrate and subsequently suppress miR-148a levels in osteoclasts, leading to reduced bone resorption and improved bone microarchitecture in osteoporotic mice with no liver and kidney toxicity [103].

Lipidoids, a class of cationic lipid-like materials, have been developed as an ideal tool for gene delivery. Lipidoids package genes through electrostatic interactions and form spherical nanoparticles 300 nm in size. Lipidoids can convey various therapeutic biomacromolecules including miRNA into target cells with high efficiency and few side effects [104, 105]. To apply lipidoid-based miRNA delivery technology for bone regeneration, Sui et al. [106] synthesized 12 candidate nanolipidoids and finally screened out a perfect biodegradable lipidoid which successfully delivered miR-335-5p into C3H10t1/2 cells and BMSCs to improve mineralized nodule formation *in vitro*. With the aid of silk scaffolds, both lipidoid-miRNA-335-5p formulation (LMF-335) and LMF-335 engineered BMSCs enhanced the healing of critical-size calvarial bone defects in mice [106].

#### 5.2.2. Polymer-Based Delivery Systems

Synthetic or natural polymer-based vectors with biodegradability, compatibility, and controlled release ability are viable alternatives for oligonucleotide delivery. In comparison with lipid-based vectors, polymer-based particles are more flexible due to their versatility in molecular size, composition, conjugations, and molecular structure [2]. Several studies have shown that polyplexes with a nanoparticle size between 10 nm and 100 nm are optimal for delivering a variety of cargo, including miRNA mimics and anti-miRs.

Polyethylenimine (PEI) is one of the cationic polymers most successfully used for gene delivery. The positive surface charge provided by the high density of amino groups makes PEI condense and protect anionic molecules, such as nucleic acids [91]. Polyplexes formed via electrostatic interactions between PEI and nucleic acids still retain a net positive charge (*ζ*-potential), which promotes their uptake by target cells through binding to negatively charged polysaccharides located outside the cell membrane [19]. Furthermore, PEI polyplexes can buffer the acidic endosomal environment through the so-called proton sponge effect, assisting oligonucleotide escape from endosomes [44]. PEI polyplexes combined with iron oxide magnetic nanoparticles (MNP) via biotin-streptavidin connections showed high uptake efficiency (~75%) and moderate cytotoxicity and provided a long-term effect when delivering miR-335 into human MSCs (hMSCs) *in vitro* [107]. Compared with commercially available magnetic vectors (Magnetofectamine, CombiMag), the miR/PEI/MNP complex formulations are not inferior with respect to miRNA uptake rates and cytotoxic effects, implying their great potential for *in vivo* therapeutic miRNA delivery to treat degenerative bone diseases as a result of MSC dysfunction [108]. Pan et al. [109] described a delivery system for miR-29b using PEI-capped gold nanoparticles (AuNPs) that exerted no obvious cytotoxicity on hMSCs and impelled miR-29b to enter the cytoplasm effectively. Compared to Lipofectamine RNAi MAX/miR-29b complexes, AuNPs/miR-29b were more efficient in improving osteoblastic differentiation and mineralization of hMSCs for the long term. Another delivery system [110], PEI-functionalized graphene oxide complex containing miR-7b overexpression plasmid (GO-PEI-miR-7b), also had excellent transfection efficiency and acceptable cytotoxicity. GO-PEI-miR-7b significantly increased bone mass, bone volume, and bone vascularization of osteoporotic OVX mice through preventing preosteoclasts from fusing into osteoclasts, which enhanced the secretion of PDGF-BB and increased the CD31^hi^Emcn^hi^ cell number.

Polyurethane (PU), a polymer composed of organic units joined by carbamate (urethane) links, is emerging as a perfect drug container owing to its good biological properties and excellent mechanical flexibilities. When modified by the acidic peptide Asp^8^, PU nanomicelles achieved the ability to efficiently encapsulate anti-miR-214 and preferentially deliver them into OSCAR^+^ osteoclasts at the bone resorption surface via the tail vein, resulting in improved bone microarchitecture and increased bone mass in OVX mice without overt toxicity or eliciting an immune response [111]. When modified by osteoblast-targeting peptide (Ser-Asp-Ser-Ser-Asp, SDSSD) which had binding affinity for periostin, PU nanomicelles selectively delivered anti-miR-214 not only to the bone formation surface but also into osteoblasts [112]. SDSSD-PU-anti-miR-214 treatment in OVX mice reduced the levels of miR-214 in osteoblasts by 80% and induced significantly increased BMD and mineral apposition rate, showing the great promise in treating the osteoblast-induced bone loss as an anabolic strategy.

Chitosan (CS) is a natural polysaccharide derived from partial deacetylation of chitin. In recent years, CS-based carriers have become an especially attractive option for gene delivery because of its low toxicity, low immunogenicity, excellent biodegradability, favorable biocompatibility, and cationic nature [113]. Its high positive charge boosts the formation of polyelectrolyte complexes with anionic siRNA or miRNA via electrostatic interactions. In addition, the hydroxyl and amino groups of CS allow for chemical conjugation of specific ligands tailored for targeted therapy. CS-tripolyphosphate (TPP) nanoparticles (at a CS-to-TPP weight ratio of 6 : 1) [114] were used to deliver the modified has-miR-199a-5p agomiR plasmid to stably overexpress miR-199a-5p, and the treatment of CS nanoparticles/199a-5p agomiR in fibrin gel improved the in situ bone regeneration of tibial defects in Sprague-Dawley (SD) rats. Pharmacological administration of a miR-34a mimic [115], miR-27a mimic [116], or miR-182 inhibitor [117] using a CS nanoparticle vector (at a CS-to-TPP weight ratio of 3 : 1) [113] attenuated postmenopausal osteoporosis of OVX mice through reducing bone resorption. The addition of hyaluronic acid (HA) to CS-based nanoparticles can loosen the tight binding between the gene and the carrier and reduce their nonspecific attachment with serum proteins, resulting in facilitated intracellular release of gene and prolonged circulation time of the delivery system [118]. Thus, Wu et al. [119] developed the CS/TPP/HA (CTH) nanoparticles to deliver naked anti-miR-138 to rat primary BMSCs. CTH/anti-miR-138 nanoparticles showed a high transfection efficiency (nearly 70%) with no cytotoxicity and significantly enhanced osteogenesis of MSCs under the condition where the highest loading efficiency was obtained at an optimum N/P ratio (20 : 1). CS/TPP/HA nanoparticle-based miRNA delivery can also be used in cell sheet technology. CS/TPP/HA nanoparticles loaded with miR-21 mimics [120] were cross-linked onto the surfaces of culture plates with 0.2% gel solution to form miR-21-functionalized culture plates where the isolated hBMSCs were seeded and induced into cell sheets based on a vitamin C-rich method. The modified hBMSC sheet showed enhanced osteogenic activity and may be a promising bone regeneration material for clinical use.

#### 5.2.3. Other Nonviral miRNA Delivery Systems

Liu et al. [121] fabricated a nonviral miRNA delivery method based on nanocapsules, which are neutrally charged and size-homogeneous with enhanced miRNA stability and high transfection efficiency and can release the miRNAs once inside cells. The nanocapsules were synthesized by in situ polymerization. The negatively charged miRNA molecules bound the positively charged monomer N-(3-aminopropyl)methacrylamide (APM) through electrostatic interactions and subsequently united the acid-degraded crosslinker ethylene glycol dimethacrylate (EGDMA) and neutral monomer acrylamide (AAM) through hydrogen bonding. Not long afterward, using this kind of nanocapsule as the building block, Meng et al. [122] developed a biodegradable miR-29b coating, the O-carboxymethyl chitosan (CMCS) coating containing miR-29b mimic nanocapsules (CMCS/n(miR-29b)) suitable for cell adhesion and growth, to modify the titanium surface. In addition, compared with the NC-miR nanocapsule titanium group, the rats treated with CMCS/n(miR-29b)-coated titanium rods showed a higher calcification rate and improved bone formation around alloplastic graft material.

CPPs, small peptides typically comprising 5-40 amino acid residues, have the ability to facilitate the intracellular translocation of a wide range of molecular cargoes covalently or noncovalently conjugated with them, including miRNAs and siRNAs [123]. Direct transduction and endocytosis have been reported to be the two main delivery mechanisms for CPP-based cellular uptake [124]. Suh et al. [125] developed a nontoxic, arginine-rich CPP called the low molecular weight protamine (LMWP). LMWP transduced the synthetic double-stranded miR-29b mimic directly into hMSCs with high transfection efficiency (6.5-fold higher than Lipofectamine® RNAi MAX after 5 h of treatment) and induced osteogenic differentiation through remarkably downregulating the gene expression of antiosteogenic factors, including HDAC4, CTNNBIP1, and DUSP2.

Aptamers are a class of artificial single-stranded oligonucleotides that can bind to specific targets (e.g., proteins, siRNA, miRNAs, and even whole cells) by folding into certain secondary or tertiary structures [126, 127]. They are usually selected from pools of artificial random-sequence oligonucleotides via screening the systematic evolution of ligands by exponential enrichment (SELEX) [128] to ensure high affinity. Moreover, they exhibit high sensitivity and selectivity due to the remarkable flexibility and convenience in the design of their structures. Based on these features, aptamers, considered as “chemical antibodies,” are emerging as a useful target delivery vector for a broad range of agents including miRNAs [129]. Liang et al. [130] screened out an aptamer (CH6) by cell-SELEX that, with the assistance of lipid nanoparticles (LNPs) as the siRNA carriers, directly delivered osteogenic *Plekho1* siRNA into osteoblasts without accumulating in osteoclasts, hepatocytes, Kupffer cells, and PBMC *in vivo*. The stable, osteoblast-specific downregulation of *Plekho1* over long periods of time (~12 d) boosted by CH6-LNPs encapsulating *Plekho1* siRNA (CH6-LNPs-siRNA) treatment led to a better-organized bone microarchitecture and increased bone mass in both osteopenic and healthy rats. miR-188, highly expressed in BMSCs from aged mice and humans, was found to regulate the age-related switch of MSCs between osteoblast and adipocyte differentiation [131]. An aptamer-antagomiR-188 targeting system succeeded in selectively silencing miR-188 in BMSCs, which increased bone formation and decreased bone marrow fat accumulation in aged mice [131]. In addition, the injection of the endothelium-specific aptamer-agomiR-195 system in aged mice enhanced CD31^hi^Emcn^hi^ vessel formation and reversed age-related osteoporosis in mice [132]. However, despite the great technological developments made in recent years, many challenges, such as rapid renal filtration, metabolic instability, and polyanionic effects, still need to be solved before the clinical translation of aptamers [129].

Extracellular vesicles (EVs) are a family of natural endosomal-derived vesicles released by numerous types of cells, including exosomes and ectosomes/macrovesicles. They influence the function of the recipient cells or tissues through transporting functional proteins, miRNAs, and mRNA from their donor cells [133]. EVs show great potential to function as targeted miRNA delivery vectors because of their nanosize, biocompatibility, bioabsorbability, low toxicity, and low immunogenicity [134]. The most attractive advantages are that EVs are sufficiently stable to travel through the body, even given orally, without the help of other biological materials, and they can naturally recognize and enter the specific recipient cells via their surface ligands. Oral administration of bovine milk-derived EVs carrying immunoregulatory miRNAs (miR-30a, -223, and -92a) and milk-specific beta-casein and beta-lactoglobulin mRNA attenuated arthritis in both *IL-1Ra*^−/−^mice and collagen-induced arthritic mice [135]. Emerging studies show that miRNA-containing EVs play an important role in bone metabolism. A delivery system [136] comprising hMSCs-derived EVs containing miR-196a and HyStem-HP hydrogel (Glycosan BioSystems, USA) stimulated bone formation and enhanced bone repair in SD rats with calvarial defects. After intravenous injection, PKH67-labelled exosomes produced by primary osteoclasts isolated from osteoclast-specific miR-214-3p knock-in mice transferred miR-214-3p to ALP+ cells (osteoblasts) and inhibited bone formation of 3-month-old female mice [137].

### 5.3. Scaffold-Based miRNA Delivery Systems

The scaffold-based delivery systems described in this review are mainly used for bone repair of refractory osteoporotic fractures. In addition to providing a structural support, an ideal scaffold should be bioactive and biodegradable and promote bone formation through providing requisite cues at the appropriate time, such as ECM proteins, diffusible factors, and engineered cells [34]. miRNA modulation at the implant interface provided by scaffolds has been reported to be a suitable way to enhance bone formation during the course of bone healing. Meanwhile, local changes in miRNA expression can accelerate the osteoinductive property of the scaffolds. Delivery from scaffolds offers more controlled and prolonged transgene expression compared with bolus delivery. Furthermore, scaffolds avoid detrimental systematic side effects of both the viral and nonviral vectors, including the unnecessary degradation and immune reaction [138]. Thus, scaffold-based miRNA delivery systems have attracted enormous attention and become an ideal resource for therapeutic application in bone tissue engineering. To date, many studies about scaffold-based miRNA delivery systems applied in bone fracture healing have been conducted to encourage their clinical implementation, and all of these systems are listed in Table 1.

Poly(ethylene glycol) (PEG), a biocompatible polymer widely investigated in bone tissue engineering, exhibits limited interactions with positively charged RNA-PEI nanoparticles due to its nonionic nature [139]. Nguyen and his coworker manufactured a kind of in situ forming PEG hydrogel system for localized and sustained delivery of RNAi to hMSCs, and presentation of miRNA-20a to hMSCs by this delivery system enhanced osteogenic differentiation of hMSCs *in vitro* and improved bone regeneration in rat calvarial defects [140]. Poly-*ϵ*-caprolactone (PCL) is a biocompatible and poorly water-soluble polymer with low melt viscosity and a slow degradation rate, making it suitable and attractive for use in hASC-assisted bone tissue engineering applications [141]. An implantation material composing PCL scaffolds, hASCs, and photoactivated miRNA-148b-silver nanoparticle (PC-miR-148b-SNP) conjugates significantly enhanced bone repair of critical-sized calvarial defects in mice, filling the defect area by 32.53 ± 8.3% in 12 weeks [142]. Polymeric nanofibers are also attractive candidates for support delivery of cells and bone-anabolic reagents in bone tissue engineering, because they closely parallel natural ECM morphology and have a microporous structure with a high surface-area-to-volume ratio [143]. The efficient release of the miR-29a inhibitor from gelatin nanofibers was sustained for at least 72 h, and the localized transient delivery of the miR-29a inhibitor enhanced ECM deposition of the preosteoblastic murine MC3T3-E1 cell line and primary BMSCs from transgenic pOBCol3.6cyan reporter mice [144].

## 6. Conclusion

An increasing number of studies have highlighted the broad and important effects of miRNAs in regulating the function and differentiation of osteoblasts, osteoclasts, BMSCs, and ECs. Without a shadow of a doubt, miRNA-based therapeutics show immense potential to treat osteoporosis and osteoporotic fractures, but only a few preclinical examples have demonstrated that miRNA treatments are successfully delivered *in vivo* into bone tissue through direct injection (Table 2). The major stumbling block is that miRNA modulators are too fragile to defend against nucleases, and they lack the targetability to specific tissues and cells *in vivo*. In this respect, the safe, effective, and receivable delivery systems and the optimized chemical modifications for miRNA modulators are the keys to resolving the problem.

AntagomiRs are the most widely investigated chemical modified anti-miRs that work *in vivo* through direct injection without the help of carriers, indicating that antagomiRs are an excellent tool for studies on the function of miRNAs in regulating bone homeostasis. However, the high dose (~80 mg/kg) required for effective inhibition impedes their clinical application. LNA-anti-miR-122 (Miravirsen, Santaris Pharma) is the first miRNA-targeted drug which has entered clinical trials and has achieved the expected results. Although it was developed to treat HCV infection, its design principles can be applied to explore desired chemically modified miRNAs for bone diseases.

Among the viral-based vectors, AAVs are one of the most reasonable options for miRNA delivery. They are small in size, nonpathogenic to humans, and low immunogenic. Most importantly, different serotypes of AAVs can infect different tissues with relative specificity. The approval of AAV-based gene therapy for lipoprotein lipase deficiency in Europe shows that AAV vectors are enforceable in clinical use with a high benefit-to-risk ratio. Despite the fact that few studies have employed AAVs for osteoporosis and osteoporotic fracture treatment, we have reason to believe that AAV-based miRNA delivery is worth further investigation. Baculovirus is another promising viral gene vector. The most desired advantages of baculoviruses include its defective replication nature and spontaneous degradation of the DNA in mammalian cells. In addition, the high transduction efficiencies in BMSCs and ASCs make baculovirus-based miRNA delivery systems especially suitable for bone regeneration materials.

Compared with viral vectors, nonviral particles are safer and less expensive. MRX34 [145], a liposomal miR-34 mimic for anticancer therapy, is another miRNA-based drug entering clinical trials in addition to Miravirsen, indicating that nonviral vectors are a viable approach for miRNA delivery in clinic to treat bone disorders. However, their intrinsic limitations including the relatively high cytotoxicity, low transfection efficiency, and poor tissue specificity need to be overcome for most clinical applications. Adding bone tissue target peptides to lipoplexes, such as (AspSerSer)6 and Asp8, offers a very good train of thought and strategy for compensating the issue of poor tissue specificity. The combined application of polyplexes and specific nanoparticles (such as MNPs and AuNPs) seem to be an ideal solution to increase the transfection efficiency and reduce the cytotoxicity.

The scaffold-based miRNA delivery has shown particular promise for bone repair of refractory osteoporotic fractures. In addition to function as a structural support, the scaffold-based delivery system can also provide a suitable environment for bone tissue regeneration through carrying growth factors and engineered stem cells and maintaining a high local and sustained concentration of therapeutic miRNAs. Enhancing biological stability and developing precise targeting capacity are the challenges needing to be addressed and resolved before applying miRNA scaffold-based therapies in clinical settings.

EVs have favorably endowed advantages for miRNA-based gene therapy. They are natural bioabsorbable gene carriers which can properly flow through the circulatory system and accurately recognize the target tissues or cells via their own unique mechanisms. Meanwhile, EVs are more easily accepted by patients because they are sufficiently stable for long-term storage and oral administration. Although at the present moment few studies have discussed the therapeutic effects of EVs encapsulating miRNAs for osteoporosis and osteoporotic fractures, EVs will become the optimal vector after the production and operation mechanisms are fully understood.

## Figures and Tables

**Table 1:** Scaffold-based miRNA delivery systems for bone tissue engineering applications.

Material	miRNA modulator	Function cell	Function	Reference
Silk scaffolds	LMF-miR-335-5p	mBMSCs	Enhance calvarial bone healing	[106]
Decalcified bone matrix	Lenti-miR-383 inhibitor	Rat BMSCs	Enhance bone formation *in vivo*	[146]
Gelatin scaffolds	Bac-miR-214 sponge	OVX-BMSCs	Heal the defect and ameliorate the bone quality	[83]
Polymeric gelatin nanofibers	miR-29a inhibitor	mBMSCs	Increase ECM deposition	[144]
Fibrin gel	CS nanoparticles/agomiR-199a-5p	Rat BMSCs	Enhance repair of tibia defects in rats	[114]
Collagen-nanohydroxyapatite scaffolds	AntagomiR-133a	hMSCs	Enhance osteogenesis	[147]
Collagen-nanohydroxyapatite scaffolds	AntagomiR-16	hMSCs	Increase the mineral calcium deposition	[148]
Poly(sebacoyl diglyceride) (PSeD) scaffold	Lenti-miR-135	Rat ADSCs	Repair the critical-sized calvarial defects in rats	[149]
Poly(glycerol sebacate) (PGS) scaffolds	Lenti-miR-31 inhibitor	Rat BMSCs	Repair the critical-sized calvarial defects in rats by 60%	[150]
PEG hydrogels	miR-20a	hMSCs	Enhance repair of the critical-size calvarial bone defect in rats	[140]
PCL scaffolds	PC-miR-148b-SNP conjugates	hASCs	Heal the critical-sized calvarial defects by 32.53 ± 8.3%	[142]
Disk-shaped poly(lactide-co-glycolide) PLGA scaffolds	Bac-BMP-2/miR-148b	hASCs	Accelerate and potentiate the bone healing and remodeling in nude mice	[84]
NF poly(L-lactic acid) scaffold that attaches the PLGA microspheres containing the HP/miRNA polyplexes	miR-26a	Endogenous stem and progenitor cells	Induce the regeneration of calvarial bone defects in healthy and osteoporotic mice	[151]
Titanium-based strontium-substituted HA	miR-21 nanocapsules	Not clear	Improve bone remodeling and osseointegration	[152]
PLGA sheets coating the surface of titanium implant	AntagomiR-204 conjugated with gold nanoparticles	Rat BMSCs	Promote osseointegration of the tibia fracture in T2DM male SD rats	[153]
Microporous titanium implants with oxide surface formed by microarc oxidation	Anti-miR-138/miR-29b lipoexes	Rat BMSCs	Enhance osteogenic activity	[154]
HyStem-HP™ hydrogel	AgomiR-26a	hBMSCs	Completely repair the critical-size calvarial bone defect and increase vascularization accordingly	[155]
HyStem-HP hydrogel	EVs containing miR-196a	hMSC	Stimulate bone formation in SD rats with calvarial defects	[136]
Dual-crosslinked photodegradable hydrogels	miR-20a/PEI complexes	hMSCs	Induce hMSC osteogenesis	[156]
*β*-Tricalcium phosphate (*β*-TCP) scaffolds	Lenti-as-miR-31	Rat ASCs	Improve the repair of critical-size calvarial bone defect in rat	[157]
*β*-TCP scaffolds	Lenti-miR-210-3p	BMSCs	Almost fully repair the critical-sized load-bearing bone defects	[158]
*β*-TCP scaffolds	Lenti-miR-26a	BMSCs	Enhance the repair of cranial bone defects in mice	[159]
HA/TCP scaffolds	Lenti-miR-216a	hAMSCs	Enhance bone formation	[160]
HA/TCP scaffolds	Lenti-miR-405b	hADSCs	Enhance bone formation *in vivo*	[161]

**Table 2:** *In vivo* miRNA-based gene therapy for osteoporosis and bone regeneration via direct injection.

miRNA modulator	Type of treatment	Mode of administration	Target cell	Mouse model	Reference
AgomiR-503	Tail vein injection	10 nmol/per mouse on days 1-3 for 3 consecutive weeks	Osteoclast	OVX mice	[63]
Agomir miR-145	Tail vein injection	100 mg/kg twice per week for 6 weeks	Osteoclast	OVX mice	[162]
AntagomiR-148a	Tail vein injection	On days 1-3 in the first and fourth weeks	Osteoclast	OVX mice	[53]
AntagomiR-103a	Tail vein injection	80 mg/kg daily on days 1-3 in the first and third weeks	BMSC	HU mice	[55]
AntagomiR-31a-5p	Periosteal injection	20 *μ*l 1 *μ*M twice per month for 3 months	Osteoclast, BMSC	Aged SD rats	[54]
miR-29b-3p-expressing plasmid	Tail vein injection	At week 2 post fracture using a microbubble-ultrasound system	mBMSCs	Femoral fracture mice	[163]
Lenti-miR-29a precursor	Tail vein injection	0.2 ml 5 × 10^9^ plaque-forming units/ml lentivirus suspension	BMSC, BMM	GC-treated rats	[74]
Lenti-pre-miR-429	Subcutaneous injection	0.1 ml 1 × 10^9^ TU/ml injected into the region of a local fracture	Osteoblast	Mice with bone fracture	[75]
Invivofectamine 3.0-miR-451a mimic	Tail vein injection	7 mg/kg on the first, second, and third days of the first, third, and fifth weeks after the ovariectomy	Osteoblast	OVX mice	[94]
Invivofectamine 2.0-miR-451 antagomiR	Not clear	7 mg/kg on day one through three during the first, third, and fifth weeks after the ovariectomy	Osteoblast	OVX mice	[164]
(D-Asp8)-liposome-antagomiR-148a	Intravenous injection	8 mg/kg once per week for 6 weeks	Osteoclast	OVX mice	[103]
(AspSerSer)6-liposome-antagomiR-214	Tail vein injection	10 mg/kg every two weeks for TG214 mice and OVX mice or once a day for 3 days before HU suspension	Osteoblast	TG214, OVX, and HU mice	[100]
(AspSerSer)6-liposome-agomiR-33-5p	Tail vein injection	Once a day for 3 days before HU suspension	Osteoblast	HU mice	[101]
Silk scaffold + LMF-335-5p local delivery	Subcutaneous injection	Twice weekly for 4 weeks	BMSC	Mice with calvarial bone defects	[106]
GO-PEI-miR-7b	Intraperitoneal injection	1 mg/kg or 10 mg/kg three times per week for 4 weeks	CD31^hi^Emcn^hi^ cell	OVX mice	[110]
Asp8-PU–anti-miR-214	Tail vein injection	16 mg/kg at an interval of 1 week for 1 month	Osteoclast	OVX mice	[111]
SDSSD-PU-anti-miR-214	Tail vein injection	10 mg/kg at an interval of 1 week for 1 month	Osteoblast	OVX mice	[112]
CS nanoparticles/miR-34a mimic	Intravenous injection	5 *μ*g or 10 *μ*g/mouse twice per week for 4-5 weeks	Osteoclast	OVX mice	[115]
CS-nanoparticles/miR-182 inhibitor	Intravenous injection	5 *μ*g/mouse twice per week for 5 weeks	Osteoclast	OVX mice	[117]
miR-27a-carrying CS nanoparticles	Intravenous injection	5 mg/mouse twice a week for 8 weeks	Osteoclast	OVX mice	[116]
BMSC-specific aptamer-antagomiR-188	Intra–bone marrow injection	40 *μ*l 0.3 *μ*M twice per month for 3 months	BMSC	Aged mice	[131]
EC-specific aptamer-agomiR-195	Tail vein injection	40 *μ*l 0.3 *μ*M once per week for 12 weeks	BMEC	Aged mice	[132]
